# Quantitative classification of pediatric swallowing through accelerometry

**DOI:** 10.1186/1743-0003-9-34

**Published:** 2012-06-09

**Authors:** Merey Celeste, Kushki Azadeh, Ervin Sejdić, Glenn Berall, Tom Chau

**Affiliations:** 1 Institute of Biomaterials and Biomedical Engineering, University of Toronto, Toronto, Ontario, Canada; 2 Bloorview Research Institute, , 150 Kilgour Road, Toronto, Ontario, Canada; 3 Department of Paediatrics, North York General Hospital, 4001 Leslie St. Toronto, Ontario, Canada; 4 Department of Electrical and Computer Engineering, University of Pittsburgh, Pittsburgh, PA, USA

**Keywords:** Swallowing, Dysphagia, Dual-axis, Accelerometry, Classification

## Abstract

**Background:**

Dysphagia or swallowing disorder negatively impacts a child’s health and development. The gold standard of dysphagia detection is videofluoroscopy which exposes the child to ionizing radiation, and requires specialized clinical expertise and expensive institutionally-based equipment, precluding day-to-day and repeated assessment of fluctuating swallowing function. Swallowing accelerometry is the non-invasive measurement of cervical vibrations during swallowing and may provide a portable and cost-effective bedside alternative. In particular, dual-axis swallowing accelerometry has demonstrated screening potential in older persons with neurogenic dysphagia, but the technique has not been evaluated in the pediatric population.

**Methods:**

In this study, dual-axis accelerometric signals were collected simultaneous to videofluoroscopic records from 29 pediatric participants (age 6.8 ± 4.8 years; 20 males) previously diagnosed with neurogenic dysphagia. Participants swallowed 3-5 sips of barium-coated boluses of different consistencies (normally, from thick puree to thin liquid) by spoon or bottle. Videofluoroscopic records were reviewed retrospectively by a clinical expert to extract swallow timings and ratings. The dual-axis acceleration signals corresponding to each identified swallow were pre-processed, segmented and trimmed prior to feature extraction from time, frequency, time-frequency and information theoretic domains. Feature space dimensionality was reduced via principal components.

**Results:**

Using 8-fold cross-validation, 16-17 dimensions and a support vector machine classifier with an RBF kernel, an adjusted accuracy of 89.6% ± 0.9 was achieved for the discrimination between swallows with and with out airway entry.

**Conclusions:**

Our results suggest that dual-axis accelerometry has merit in the non-invasive detection of unsafe swallows in children and deserves further consideration as a pediatric medical device.

## Background

Feeding disorders encompass a broad range of problems associated with eating solid and liquid foods. Difficulty with the process of swallowing is known as dysphagia, and can occur in both adult and pediatric populations [1]. Epidemiologic data on the prevalence of dysphagia in children is not readily available; however feeding disorders as a whole are estimated to be present in a significant and increasing portion of the pediatric population: in 25% to 45% of typically developing children and in 33% to 80% of children with developmental disorders [2]. In neurogenic dysphagia, swallowing difficulties arise secondary to neurological impairments. Particularly, in neurological conditions such as cerebral palsy, incidence of dysphagia is high [3]. Dysphagia impacts the health and well-being of a child as the disorder may lead to malnutrition, dehydration and impairment of physical growth [4]. Dysphagia can also induce feeding-related stress and challenges, affecting the psychosocial well-being of the child, family and other caregivers [2]. A particularly dangerous condition, aspiration pneumonia, is frequently associated with dysphagia [5]. Silent aspiration, that is, the entry of foodstuffs into the airway in the absence of a cough or other overt response, is generally difficult to detect during routine clinical swallowing assessments without videofluoroscopy. Recently, Weir and colleagues reported that silent aspiration occured frequently in children with dysphagia; over 80% of their sample of 300 children aspirated silently [6]. Further, silent aspiration was significantly associated with neurological impairment.

Early evaluation by a clinical team may greatly reduce health issues that can result from dysphagia. In particular, instrumental evaluations of swallowing facilitate the visualization of the bolus trajectory and motion of anatomical structures throughout the different phases of swallowing. The current standard is the videoflouroscopic swallow study (VFSS), where the patient swallows barium coated substances of various consistencies while lateral X-ray images of the oral cavity, pharynx, larynx and upper esophagus are displayed in real-time for live viewing and recorded for subsequent review. As one part of the assessment, the clinical team discerns whether or not the bolus passes into the airway, either into the laryngeal vestibulum above the vocal chords or past the vocal chords and into the inferior airways [7].

Clinical evaluations of the health of the swallow have yielded varying levels of agreement amongst clinicians and trained experts [8]. Although perfect agreement was not achieved, expert diagnosis matched on most occasions, indicating the effectiveness of VFSS as a means of detecting unsafe swallowing. Hind et al.’s study [9] resulted in agreement rates of 93-95% in clinical diagnosis of an unhealthy swallow. More recently, Bryant et al. [8] found a higher variability and lower accuracy of detecting unhealthy swallows: between 77% and 88% agreement amongst participating clinicians.

VFSS is not without its shortcomings. Proper interpretation of the swallow usually requires an experienced practitioner or a team of assessors. The method itself also exposes children to ionizing radiation and therefore should be used minimally [7]. As well, the process is expensive both in terms of equipment and human resources. Furthermore, VFSS can only provide a snapshot of a patient’s swallowing function, despite the fact that this function can vary from day to day. Finally, many children find VFSS frightening and uncomfortable [10]. These issues have motivated a search for alternative means of instrumentally evaluating swallowing function.

Recently, the use of accelerometry has been under investigation as a non-invasive, low cost technique for characterizing swallowing [11,12]. Initial research involving the placement of an single-axis accelerometer at the thyroid cartilage to measure throat vibrations has garnered positive results in the identification of dysphagic activity within the adult population [13]. Nonetheless, Reddy et al. observed that the patient’s compliance to measurement protocol could affect the accuracy of classifying swallows [13]. Particularly, in the pediatric case, one can reasonably expect that a child may be inclined to exhibit more spontaneous vocalizations and bodily movements during the feeding protocol, in turn, generating more contaminant vibration signals. In addition, children with neurogenic dysphagia often exhibit behavioral feeding challenges (e.g., food refusal, food expulsion, disruptive mealtime routines, short attention span, impulsivity and distractibility) that further complicate the assessment of swallowing function [3]. Thus, the classification of swallowing accelerometry in the pediatric population with neurological impairment or disease likely presents further analytical challenges than those encountered in the adult case. In 2006, Lee et al. proposed a radial basis classifier for the classification of uniaxial vibrations associated with swallowing activity in children [14]. Although they achieved an 80% adjusted accuracy, their classifier only detected a very specific event, i.e., airway entry with inspiratory airflow. Dual-axis accelerometry provides unique signal information in each axis of vibration (superior-inferior and anterior-posterior) attributed to the motion of the hyoid and larynx [15,16]. Dual-axis accelerometry may serve as an informative adjunct to clinical swallow assessment, may provide a means of timely and repeatable non-invasive assessment of swallowing function (especially post-stroke) and may help to streamline referrals to videofluoroscopy, reducing unnecessary exposure to ionizing radiation and reducing radiology wait-times. The present study aims to generalize detection to any instance of airway entry in children with neurogenic dysphagia and to build upon methods of data collection, processing and classification which have proven to be effective in adult populations [12].

## Methods

### Data acquisition and swallow identification

Cervical accelerometry swallowing samples were collected from 29 patients (aged 6.8±4.8, 20 male) of the Holland Bloorview Kids Rehabilitation Hospital’s feeding clinic. Participants of the feeding clinic all had neurological conditions such as Cerebral Palsy, seizure disorder, developmental delay, brain injury and Downe Syndrome. All participants were diagnosed with probable feeding disorders at a previous appointment by the attending pediatrician through medical history and feeding patterns. At the time of the study, all participants were being fed orally. Consent to participate in the study was acquired from the parent or caregiver of the participating child. The data collection session occurred during the feeding clinic’s VFSS assessment, and followed a protocol approved by the hospital’s Board of Ethics.

The accelerometer (Analog Devices, ADXL322,) was taped to the participant’s neck at the level of the cricoid cartilage such that the sensitive axes of the accelerometer were aligned with the superior-inferior (S-I) and posterior-anterior (A-P) anatomical directions. According to the modified barium swallow procedure [17], each child was fed barium-coated boluses of varying consistencies, ranging from thick purée to thin liquid. Children were generally fed by spoon and infants were fed by bottle. The starting consistency varied by child and was determined based on the recent case history.

The swallowing vibration signals from the accelerometer were filtered and amplified (Astro-Med Inc., Grass, P55 A.C. Preamplifier; filtered 1Hz to 3 kHz; amplification 10x) and sampled at 10 kHz via a data-acquisition card (National Instruments, USB NI-6210) prior to storage on a research computer. Bolus activity in the cervical region was captured by lateral fluoroscopic video recording onto a research computer via a PCI card (National Instruments, PCI 1405). The trigger to initiate and terminate the recording of these inputs was controlled through a custom LabVIEW application (National Instruments), which served to synchronize the VFSS video recording with the accelerometric data, enabling swallow time-stamp identification retrospectively.

All recordings of the VFSS video were reviewed off-line by the feeding clinic’s pediatrician to ascertain the time-stamp of occurrence and nature of each swallow taking place. The VFSS video was played at one third speed and the point at which the bolus or accumulated residue passed below the level of the epiglottis was selected as the time of swallow. Each swallow was assigned one of three ratings, as outlined in Table 1[12].

**Table 1:** Swallow rating based on path of bolus

**Rating**	**Clinical description**
0	Material does not enter the airway
1	Material enters the airway,
	and contacts the true vocal folds but does not pass below
2	Material enters the airway,
	and passes below the true vocal folds

Each swallow was assigned a number using this system based on visual observation of the VFSS video playback.

Swallows rated as 1 were discarded from the study to avoid ambiguity between safe and unsafe swallowing. The resultant data set contained 94 swallows rated 2 (i.e. unsafe swallows) and 544 swallows rated 0 (i.e. safe swallows).

### Pre-processing

The dual axis accelerometer generated time-stamped S-I and A-P vibration signals. Each signal was processed individually with the treatments described in this section. Initially, the signal was down-sampled to 1kHz, as Lee et al. demonstrated little of the signal’s energy exists at frequencies above 100Hz [15].

Next, segments deemed to contain vocalizations by the robust algorithm for pitch tracking (RAPT) were identified [18]. The RAPT parameters were adjusted to minimise the number of discarded segments which contained swallows as follows: the maximum possible fundamental frequency was set to 1000Hz, the duration of frame size was set to 0.01s, the duration of the correlation window was set to 0.01s and the maximum number of hypotheses at each frame was set to 50. Segments identified by the RAPT algorithm with the adjusted parameters were discarded. Any swallows within these removed segments were therefore eliminated from further consideration.

Low frequency components associated with head movement were de-trended using a least-squares spline approximation [19]. Segmentation of each swallow from the accelerometric record proceeded as follows: the signal was marked at the time stamp identified via videofluoroscopy. Each segment was produced by expanding one second on either end of the time stamp identified via videofluroscopy, ensuring swallow activity was captured. Each segment was trimmed through visual and audio inspection to eliminate extraneous data from the beginning or end of each segment.

**Figure 1:**
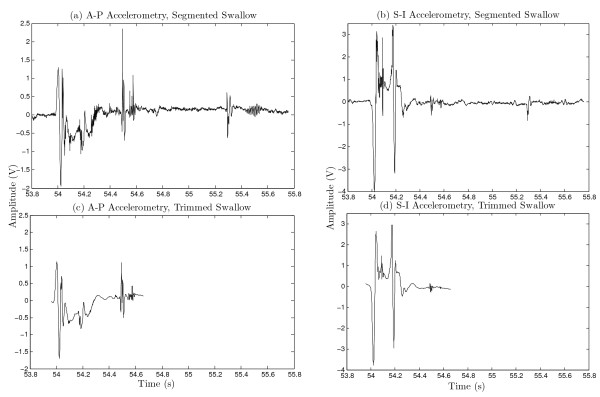
**Swallowing Accelerometry Signal.** Figures **(a)** and **(b)** display a swallowing accelerometry signal from the AP and SI axes that have been processed and segmented. Figures **(c)** and **(d)** display these signals after being trimmed and filtered.

Each segment was filtered using a denoising algorithm developed for swallowing accelerometry signals which resulted in a smaller reconstruction error than other established methods [20]. This method was based on a ten-level discrete wavelet transform using the Meyer wavelet with soft thresholding. An example of a trimmed and filtered swallow signal can be seen in Figure 1. Note that duration and visual characteristics vary between swallows.

### Feature extraction

For each trimmed segment (i.e., each swallow), features were extracted from the time, frequency, time-frequency and information-theoretic domains to capture key differences between safe and unsafe swallows. This collection, totalling 144 features, was compiled from two sources: 1) a survey of literature in swallowing accelerometry (as referenced below), and 2) visual inspection of differences in signal behavior between safe and unsafe swallows.

Based on results from the literature survey, the following features were calculated for each of the A-P and S-I signal segments: mean [12,21], variance [12,21], skewness [12,21], kurtosis [12,21], entropy rate [21], memory [21], Lempel-Ziv complexity [21], measures of the relative energy and entropy in each wavelet decomposition level as extracted from a 10-level discrete wavelet decomposition of the signal using the discrete Meyer wavelet [21] and as extracted from a 20-level Daubechies 5 wavelet decomposition [12], normality [12,14], stationarity [14], number of zero-crossings [12,14], dispersion ratio [14], interquartile range [14], peak Fast Fourier Transform (FFT) magnitude [12], frequency at spectral peak [12], maximum hyolaryngeal excursion (estimated via double integration of accelerometry [12,16]). Two features were calculated between the A-P and S-I signals: the cross-entropy rate and cross-correlation of the signals at zero lag [21].

Based on visual inspection, the following additional features were computed for each of the A-P and S-I signal segments.

From the time domain: 

· median

· the absolute difference between mean and median

· p value of the chi-squared test for normality

· total area under the main lobe: the portion of the signal corresponding to maximum hyolaryngeal excursion

· width of the main lobe

From the frequency domain: 

· frequency corresponding to maximum spectral density over all time of the short-time Fourier transform spectrogram (max frequency)

· difference between frequency values corresponding to 75% and 25% of maximum spectral density at time corresponding to max frequency

· 20 features computed by the summation of power spectral density values within a 10Hz range, from 0 to 200Hz (calculated in unit of power per radian per sample using Welch’s averaged modified periodogram method of spectral estimation)

### Feature selection and classification method

Feature extraction yielded 144 features for each of the 638 trimmed swallow samples. The number of healthy swallows largely outnumbered the alternate class, prompting the use of bootstrap re-sampling to create a balanced distribution class [22].

Classification was performed using 8 fold cross-validation. In each fold, training and test sets contained a balanced distribution of both classes. The dimensionality of the training subset was reduced using principal component analysis (PCA) with a variance threshold of 80%. The principle components served as the inputs to four different classifier models: linear discriminant analysis (LDA) using Euclidean and Mahalanobis distance measure variants, and support vector machines (SVM) trained with a linear kernel and with a Radial Basis Function (RBF) kernel. LDA was selected as a comparison point to recent studies [12] while SVM was selected for its insensitivity to overtraining and circumvention of the curse of dimensionality [23]. Adjusted accuracy was averaged over all folds and runs.

**Figure 2:**
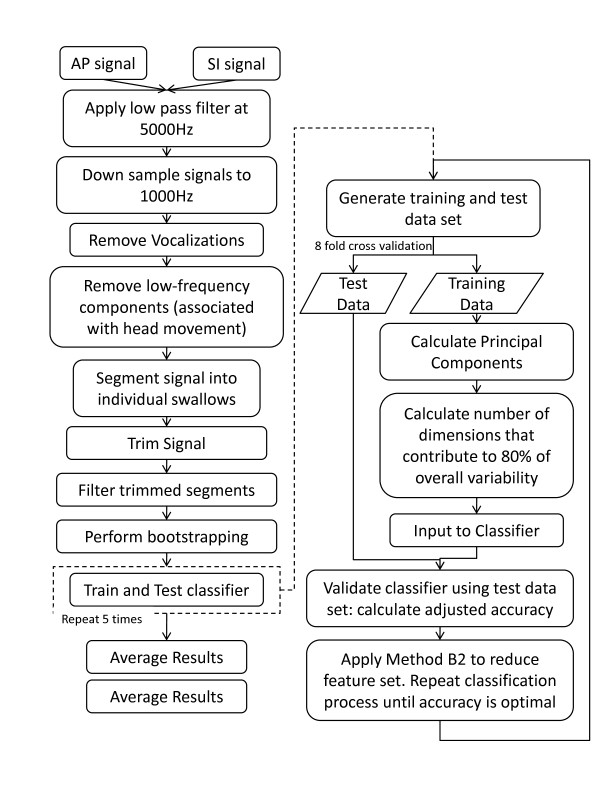
**Flowchart.** Process flow steps outlining the preprocessing, feature extraction and selection and classification techniques, as described in Methods.

The classifier was further optimized through a method of removing redundant variables for a data set composed of more than 10 features. In particular, to determine which variables may be discarded, we deployed method B2, as introduced by Joliffe [24], given its ability to parsimoniously represent the data while achieving accuracies comparable to those attained with the full feature set. Using B2, the feature set was reduced by removing variables associated with the largest coefficient in each of the last 70 principal components when ordered by their contribution to variance, from largest to smallest. The new feature subset was then input to the SVM classifier with the RBF kernel. All steps outlined in the preceding sections are outlined in Figure 2.

## Results

Table 2 displays averaged results of multiple runs of 8-fold cross-validation for each of the four different classification models using principal components comprising the full 144 feature set. The reduction in dimensionality as a result of PCA is also reported for each of the models. Overall, SVM with the RBF kernel generated the highest average adjusted accuracy, 80.6%, a value which is significantly different from the adjusted accuracies of LDA with Euclidean, LDA with Mahalanobis and SVM with a linear kernel (Wilcoxon rank sum test, p<0.001).

**Table 2:** Results

**Classifier**	**Sensitivity**	**Specificity**	**Adjusted**	**Dimensionality**
	**(%)**	**(%)**	**accuracy (%)**	
Using 144 features:				
LDA Euclidean	50.7±7.5	74.9±6.9	62.8±3.3	17
LDA Mahalanobis	69.8±2.1	51.4±4.7	60.6±1.9	16
SVM linear	51.5±2.9	72.4±2.7	62.0±2.1	16
SVM RBF	80.0±7.0	81.2±5.3	80.6±3.9	18
Using 48 features:				
SVM RBF	89.6±0.9	92.2±1.6	86.9±0.6	16

Averaged results for each classifier.

Using method B2, features calculated through wavelet decomposition (relative energy from each of the 10-levels of discrete Meyer wavelet, and the 20-level Daubechis 5 wavelet) as well as from the summation of power spectral densities were frequently associated with the largest coefficient of the last 70 components. These features were removed leaving a 48 feature subset. PCA diminished the dimensionality from 144 to 16 or 17 dimensions, depending on the particular iteration and fold. The average input dimensionality to the classifier was 17±1. Multiple runs of 8-fold cross-validation using this reduced feature set obtained an average adjusted accuracy of 89.6*%*±0.9, a sensitivity of 92.2*%*±1.6 and a specificity of 86.9*%*±0.6.

## Discussion

### Pre-processing

Methods used in this study to pre-process accelerometry signals were previously developed for an adult age group. Nonetheless, applying these methods to the pediatric realm resulted in a classifier with a high adjusted accuracy. Reasoning behind the success of translating these techniques is discussed for each of the pre-processing methods.

Removing vocalizations, including coughing noises, is an important step in developing a classifier based on swallows. The frequency of speech falls below 1000Hz, within the spectral range of swallowing accelerometry signals, and therefore, may provide false information when calculating features. The characterization of vocalizations based on age is challenging; the fundamental frequency range of vocalizations can change from day to day, and may vary among persons of the same gender and age [25]. Therefore, the pediatric participants in this study, consisting of both genders and a range of ages, likely produced vocalizations with a wide frequency range comparable to that encountered in adult studies. As a consequence, the vocalization removal scheme previously proposed for adults required only some adjustment in order to be effective with the pediatric data considered herein.

Similarly, previous adult studies did not identify an age effect on the spectral features of swallowing accelerometry in the presence of head movement [19]. These studies did however conclude that head motions introduce non-trivial low frequency components that should be detrended. Thus this technique was applied to our data.

Finally, the denoising filter, also borrowed from previous adult studies, was applicable to the pediatric signals because the algorithm optimizes the threshold value in a reduced wavelet threshold for each data set under consideration. In this way, the algorithm was customized to the pediatric signals collected in this study.

One limitation of our pre-processing research is the method of isolating the swallowing signal. Visual detection of the bolus using the VFSS recording, followed by visual/audio review of the accelerometry signal leaves room for human error. Automatic segmentation techniques are not applicable at this time; current methods do not account for the large variability in the contamination of signals found in the pediatric realm [26]. Thus, careful human analysis was required to isolate each swallow. In the future, novel segmentation algorithms ought to be developed to account for the enormity of contaminants in the pediatric case, including for example, gross postural changes, rapid limb movement and stress-induced psychogenic lacrimation.

### Feature extraction and selection

New swallowing accelerometry features were introduced and used for classification in this study, suggesting that information overlooked in previous studies was indeed necessary to attain the reported accuracies.

Considering the features related to the main lobe, Zorotto et al. correlate the peak accelerometry amplitude to the movement of the hyoid bone and larynx during a swallow [16]. Studies evaluating dysphagic swallowing suggest penetration or aspiration may occur with delay or absence of hyolaryngeal movement or improper closure of the larynx [27]. Therefore, differences in hyolaryngeal movement may result in a change in the duration and area of the main lobe, providing features which discriminate between healthy and unhealthy swallows.

The selection of features relating to the short-time Fourier transform is perhaps unsurprising given that past studies demonstrated the discriminative value of time-frequency features of swallowing accelerometry signals [12].

### Selection of principal components

The dimensionality of the principal component sub-space balanced three criteria: 

· Most of the variability of the principal component space ought to be captured. To meet this criterion, principal components contributing to 80% of the variability were selected.;

· The “curse of dimensionality” ought to be mitigated. To fulfill this criterion, the ratio of sample size to the number of dimensions was 30, which exceeds the minimum of 5 prescribed in literature [27]. [28]

· The number of principal components ought to minimize the error of the classifier. To meet this criterion, components were added until the “peaking” point [29] was reached.

These criteria were all met with the range of 16-17 selected dimensions.

Removal of the wavelet decomposition and power spectrum summation components resulted in a significant improvement to the accuracy of the classifier. Method B2 may therefore be interpreted as a noise removal technique: the last 70 principal components out of 144 minimally contributed to the overall variability. Therefore the features that provide the most influence to these insignificant principal components can be considered to be disruptive to the overall accuracy of the classifier. Indeed, removal of these features resulted in a classifier with improved accuracy.

In reducing the number of features, variables associated with the largest coefficients of the last 70 components were evenly distributed between the AP and the SI axes. Likewise, the features retained were also sourced equally from each axis. No one axis was singled out as more useful than the other, reflecting the importance of the dual axis combination. This corroborates a prior study [15] which concluded that different information was collected by the accelerometry signal of each axis, and supports the use of dual-axis accelerometry within the pediatric realm.

### Clinical implications

The results reported herein suggest that dual-axis accelerometry, being non-invasive, inexpensive and sensitive to airway entry events, may indeed be an informative adjunct to routine clinical swallowing assessment in children with suspicion of neurogenic dysphagia. A screening tool based on dual-axis accelerometry would be particularly useful in assessing children who aspirate silently. However, the analytical challenges posed by the noisy vibration signals demand that the associated swallowing protocol be as controlled as possible, a requirement that may be difficult to fulfill in the pediatric case.

### Classification

The adjusted accuracy of the SVM classifier with the RBF kernel using the reduced feature set compares favorably with results attained in classifying adult dysphagic swallows (between 74.7 and 84.2% accuracy) [12], as well as studies of inter-rater reliability of videofluoroscopic examination [8,9]. The most promising VFSS inter-rater reliability was reported by Hind et al., who analyzed swallow ratings made by a large number of clinicians from a diverse collection of hospitals against a trained standard. However, clinicians in that study underwent intensive training [9] and hence, a lower accuracy may be expected in real-world situations where standardized training is not available or practical.

The improvement in accuracy attained using the nonlinear RBF kernel indicates that the two classes were not linearly separable. A high dimensional input vector, i.e., 16 or 17 dimensions was necessitated for a number of potential reasons. Visual inspection of the accelerometer data did not reveal a regular signal pattern for either class, suggesting that no obvious low dimensional representation of the data would yield class separability. Further, the data were necessarily collected under a variety of conditions; feeding modality (spoon or cup), bolus viscosity (thin liquid to thick purée), participant age and etiology of dysphagia varied among participants. These variables may have introduced differential effects on the accelerometer signals, even within one class. Hence, a multi-dimensional, non-linear classifier may be needed to sort through such complex inputs.

### Limitations

Our sample was skewed towards the younger end of the pediatric spectrum. Hence, replication of our study with a larger sample that is more uniformly distributed in age would be a worthwhile future endeavor. As mentioned earlier, the current algorithm relies on manual segmentation of the swallowing signals. Future research should examine the applicability of automatic segmentation methods such as the quadratic variation method proposed by Damouras et al. [30] for swallowing signals. Another potential limitation of accelerometry is the reported dependence of signal characteristics on the nature of the swallowing task [31]. This dependence might be mitigated via the administration of a standardized swallowing protocol.

## Conclusions

We presented the first study of dual-axis accelerometry for the discrimination of swallowing signals in the dysphagic pediatric population. We proposed a nonlinear classifier, which achieved an adjusted accuracy of 90% using an input vector of up to 17 dimensions. This study extends previous work conducted with an adult dysphagic population, demonstrating that while differences in input signal quality exist between adult and pediatric swallowing, dual-axis accelerometry holds significant promise in the pediatric domain.

## Competing interests

The authors declare that they have no competing interests.

## Authors’ contributions

CM performed the data collection, carried out the selection and analysis of classification and drafted the manuscript. AK advised on classification selection and analysis and edited the manuscript. ES provided insight on processing methods. GB provided clinical rating of all swallows and advised on data collection protocol. TC conceived of the study and edited the manuscript. All authors read and approved the final manuscript.
